# A Wristwatch-Based Wireless Sensor Platform for IoT Health Monitoring Applications

**DOI:** 10.3390/s20061675

**Published:** 2020-03-17

**Authors:** Sanjeev Kumar, John L. Buckley, John Barton, Melusine Pigeon, Robert Newberry, Matthew Rodencal, Adhurim Hajzeraj, Tim Hannon, Ken Rogers, Declan Casey, Donal O’Sullivan, Brendan O’Flynn

**Affiliations:** 1 Tyndall National Institute, University College Cork, Dyke Parade, T12R5CP Cork, Ireland; john.buckley@tyndall.ie (J.L.B.); john.barton@tyndall.ie (J.B.); melusine.pigeon@tyndall.ie (M.P.); adhurim.hajzeraj@tyndall.ie (A.H.); ken.rodgers@tyndall.ie (K.R.); declan.casey@tyndall.ie (D.C.); donal.osullivan@tyndall.ie (D.O.); brendan.oflynn@tyndall.ie (B.O.); 2 Sanmina Corporation, 13000 S. Memorial Parkway, Huntsville, AL 35803, USA; robert.newberry@sanmina.com (R.N.); matthew.rodencal@sanmina.com (M.R.); tim.hannon@sanmina.com (T.H.)

**Keywords:** IoT, wearables, Sub-GHz, electrically small antennas, LDS, wristwatch antenna, bandwidth enhancement, healthcare, PPG, wireless sensor

## Abstract

A wristwatch-based wireless sensor platform for IoT wearable health monitoring applications is presented. The paper describes the platform in detail, with a particular focus given to the design of a novel and compact wireless sub-system for 868 MHz wristwatch applications. An example application using the developed platform is discussed for arterial oxygen saturation (SpO_2_) and heart rate measurement using optical photoplethysmography (PPG). A comparison of the wireless performance in the 868 MHz and the 2.45 GHz bands is performed. Another contribution of this work is the development of a highly integrated 868 MHz antenna. The antenna structure is printed on the surface of a wristwatch enclosure using laser direct structuring (LDS) technology. At 868 MHz, a low specific absorption rate (SAR) of less than 0.1% of the maximum permissible limit in the simulation is demonstrated. The measured on-body prototype antenna exhibits a −10 dB impedance bandwidth of 36 MHz, a peak realized gain of −4.86 dBi and a radiation efficiency of 14.53% at 868 MHz. To evaluate the performance of the developed 868 MHz sensor platform, the wireless communication range measurements are performed in an indoor environment and compared with a commercial Bluetooth wristwatch device.

## 1. Introduction

The Internet of Things (IoT) is a network of internet-connected devices that contain embedded sensors and actuators to communicate with the external environment [1]. Because of its widespread applications in various areas, such as in healthcare, agriculture, sports and security, the number of internet-connected devices is increasing at a rapid rate [2,3]. The IoT market is growing continuously, and it is expected that, by 2025, global IoT market revenue will rise to around 1.6 trillion U.S. dollars (USD) [4]. The IoT-enabled wearable sensor devices have become a promising technology that enables applications such as continuous wireless monitoring of vital physiological parameters such as arterial oxygen saturation (SpO_2_) and heart rate (HR) [5]. Among the various wearable devices available, it is forecasted that the end-user spending on smartwatches could exceed 27 billion USD in 2021 [6].

Photoplethysmography (PPG) is a technology that uses optical sensors for non-invasive monitoring of the volume changes in arterial blood [7,8]. PPG is widely used in pulse oximetry for the measurement of various health parameters such as SpO_2_, HR, blood pressure, anaesthetic depth and respiration rate [9,10]. Some companies, such as Apple, Huawei, Fitbit and Fossil have recently added SpO_2_ monitoring features to their wrist-worn devices [11,12,13,14]. Estimating the oxygen saturation level in the bloodstream helps in determining the sleep pattern and the oxygen saturation status of a person during sports, fitness, and many other physical activities. 

Wireless communication is a key technology in continuous sensing and monitoring the health conditions of a person [15]. Generally, the sensing device has a limited computation and processing capability, and typically, an additional processing unit is incorporated for data processing [16]. Further, a series of operations such as data visualization for the end-user, and transmitting the measured data to other centers can be accomplished using a smartphone application [16,17,18]. Typically, wearable healthcare sensors and systems require constant accessibility and operation of the sensed data [16]. Advancement in cloud computing technology offers a great opportunity to transmit the sensor data to the IoT cloud, and when required, an authorized user can easily access the data with any internet-connected device [19,20,21]. 

Currently, the majority of wrist-worn devices operate in the conventional 2.45 GHz industrial, scientific and medical (ISM) frequency band [12,13,17,22,23,24]. Wireless communications at the 2.45 GHz band have several advantages, such as worldwide availability, a higher data rate, compatibility with a large number of wireless standards, and small size antenna [5]. However, the 2.45 GHz band has now become highly crowded, which can dampen the communication reliability and the quality of service (QoS). [5,25,26]. 

An emerging alternative to the 2.45 GHz ISM band for IoT applications is the Sub-GHz band [5,27]. The most popular Sub-GHz bands include 433 MHz (Asia), 868 MHz (Europe), and 915 MHz (USA) [28,29,30]. In [31], the development of a wearable tracking system using the Sigfox protocol is reported, where the implemented 868 MHz meandered inverted-F antenna requires an area of 270 × 75 mm^2^. A wearable long-range (LoRa) sensor device for tracking and wireless health monitoring is reported in [32]. However, in this study, the implemented hardware components were not developed, and instead, commercial radio and whip antenna were used. In [33], a 915 MHz LoRa-based wearable sensor platform for IoT health monitoring is reported, where a commercial antenna was implemented, and the height of the developed device is large (45 mm). In [5], the authors developed a 915 MHz wristwatch-based wireless sensor device, where a comparison of the communication performance at 915 MHz and 2.45 GHz is also discussed; however, the developed sensor device was not fully functional. To summarize, the majority of the wearable wireless sensor platforms reported in the literature use a commercial radio and antenna and require a large size for their implementation [31,32,33,34]. 

A number of Sub-GHz band wrist-worn antennas have been reported in the literature [5,35,36,37,38,39,40]. An 868 MHz helical wristband antenna reported in [35] has a −10 dB impedance bandwidth of 17 MHz but has a low peak realized gain of −13 dBi. A dual-band wrist-worn loop antenna reported in [36] has a −10 dB impedance bandwidth of 180 MHz; however, it has a low radiation efficiency of 6.4%. Furthermore, a wrist-worn dipole antenna reported in [37] has a wide impedance bandwidth of 160 MHz; however, this antenna has a low radiation efficiency of 6.3% at 900 MHz. The authors present a 915 MHz wristwatch planar inverted-F antenna (PIFA) in [40]. This antenna has an on-body measured −10 dB impedance bandwidth of 26.4 MHz, a peak realized gain of −0.57 dBi and a radiation efficiency of 46.8% at 915 MHz. However, the height of the antenna from the nearest ground plane is large (10.3 mm) when compared with other designs reported in the literature [5,35,36,37,38,39,40]. In [5], the authors present a 915 MHz meandered PIFA wristwatch-integrated antenna. This antenna shows an on-body peak realized gain of −6.1 dBi and a −10 dB impedance bandwidth of 55 MHz. The majority of Sub-GHz band wrist-worn antennas reported in the literature do not exhibit satisfactory impedance and radiation performance simultaneously [35,36,37,38,39]. In addition, most of the reported antennas (except [5]) assume an ideal environment and do not take into account the effects of the nearby internal components of the wristwatch device. 

The key focus of this work is to develop a compact wireless sub-system for 868 MHz and a gateway to establish wireless communication between the wristwatch sensor platform and a smartphone. The novel aspect of this paper includes the design of a compact on-body 868 MHz antenna and a radio that fit into the developed wristwatch-based wireless sensor platform. In contrast to other research efforts reported in the literature, the developed 868 MHz antenna includes all the internal components of the practical wristwatch sensor device and focuses on achieving improved impedance and radiation characteristics. In addition, this work also evaluates the wireless communication range performance of the developed wristwatch sensor platform in an indoor environment and demonstrates a greater communication range when compared with a commercial Bluetooth (BLE) wristwatch device. 

The remainder of the paper is organized as follows: a comparison of wireless performance in the 868 MHz and 2.45 GHz ISM bands is discussed in Section 2. The system design aspect of the developed sensor platform is described in Section 3. In Section 4, the antenna design, fabrication process, and measured results are described. The communication range measurements of the developed sensor platform are presented in Section 5. Section 6 presents the sensor platform implementations and clinical trial settings. Finally, in Section 7, the main conclusions and future research plans are summarized. 

## 2. Comparison of Wireless Performance in the 868 MHz and 2.45 GHz ISM Bands

Based on the literature and numerical calculations, a comparison between the wireless performance in the 868 MHz and 2.45 GHz ISM bands is outlined in this section. 

### 2.1. Free Space Path Loss

**Figure 1:**
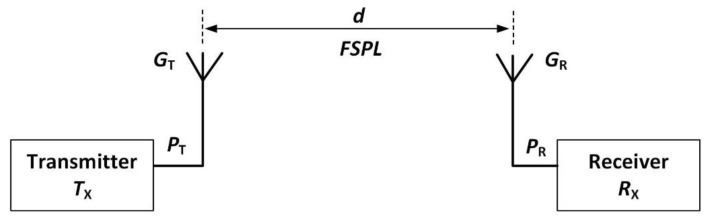
Representation of the transmitter and receiver setup for FSPL calculations.

Free space path loss (*FSPL*) is a function of the free-space wavelength (λ_0_) of the radio signal and the separation between two antennas, as shown in Figure 1. The term *T*_X_ represents the transmitter, and *R*_X_ represents the receiver, and the separation between the two is denoted as *d*. The transmitted power is denoted as *P*_T_, the received power is denoted as *P*_R_, and the gain of the transmitting and receiving antenna is denoted as *G*_T_ and *G*_R_, respectively. 

The *FSPL* is given as

(1) $$FSPL\left(\mathrm{dB}\right)=10\;log{\left(4\pi d/\lambda_{0}\right)}^{2}$$

The free-space wavelength, *λ*_0_ at *f*_0_ = 868 MHz is 0.345 m, and at 2.45 GHz, *λ*_0_ = 0.122 m. Assuming the separation (*d*) between transmit and receive antenna to be 3 m, the *FSPL* from Equation (1) is calculated as 40.8 dB at 868 MHz and 49.8 dB at 2.45 GHz. Thus, lowering the frequency of operation from 2.45 GHz to 868 MHz, the *FSPL* is decreased by 9 dB, i.e., the transmitted radio signal experiences 9 dB lower attenuation in the 868 MHz band compared to the 2.45 GHz band.

### 2.2. Radio Frequency (RF) Attenuation in Indoor Environment

The propagation of radio frequency (RF) signals through materials is highly dependent on the frequency of operation [41]. When compared with the 2.45 GHz band, improved RF signal penetration through walls, building, and other obstacles at the lower frequency is a definite advantage of the Sub-GHz band [41,42,43]. For example, an RF signal propagating through an 8” concrete wall experiences 7 dB more attenuation at 2.45 GHz than 1 GHz, i.e., a system operating at 1 GHz will have a propagation range of more than twice as far in comparison to the 2.45 GHz band [42]. 

### 2.3. Co-Existence Issues 

Currently, several wireless solutions such as Bluetooth, W-Fi, ZigBee, and other protocols operate in the license free 2.45 GHz band, and this leads to the co-existence issue. The co-existence of several wireless standards centred around the 2.45 GHz band degrades the wireless performance of the devices in terms of data packet loss and transmission delay [25,26]. Applications such as healthcare require high reliability, and therefore a technique to overcome or reduce the issue of co-existence is required. It is shown that Sub-GHz bands such as 868 MHz and 915 MHz bands are less congested and can circumvent the co-existence issues of the 2.45 GHz band, and can improve the QoS [27,44].

### 2.4. Power Consumption

Power consumption is a critical concern for battery-powered wearable devices, and it depends on several factors such as the wireless protocol, radio transceiver, frequency of operation, and the channel co-existence [5,45]. Due to the co-existence issue at the 2.45 GHz band, multiple retransmissions may be required, which leads to an increased power consumption [25,26]. Due to less attenuation of RF propagating through walls and other obstacles, the radio transceiver operating at 868 MHz requires less power to achieve a similar communication range as at 2.45 GHz [5,41,43]. In [5], the current consumption for 915 MHz and 2.45 GHz bands are analytically calculated and show that, depending on the chosen sampling rate and applications, the Sub-GHz band sensor devices have the potential to operate at a significantly lower current level than the 2.45 GHz band.

### 2.5. Wireless Communication Range

In this sub-section, the free-space wireless communication range calculations are performed for an 868 MHz radio transceiver (AT86RF212B) used in this work [46], and for a BLE device where adaptive frequency hopping is not enabled. BLE is a common choice for a communication protocol for sensors due to its low power consumption, low cost and ease of deployment. The communication range of a wireless device is governed by the Friis’ transmission equation for free-space propagation. As illustrated in Figure 1, the Friis’ transmission equation relates the received and transmitted power between two antennas separated by distance d and is given as [47]

(2) $$P_{R}\left(\mathrm{dBm}\right)=P_{T}\left(d\mathrm{Bm}\right)+G_{T}\left(\mathrm{dBi}\right)+G_{R}\left(\mathrm{dBi}\right)+10\;log{\left(\lambda_{0}/4\pi d\right)}^{2}$$

In Europe, the maximum effective radiated power limit at 2.45 GHz for the BLE devices (for example, BLE 4.0) is 10 dBm, and at 868 MHz, the limit is 14 dBm [48,49,50]. In Table 1, the parameters required to calculate the theoretical free-space wireless communication range at 868 MHz and BLE 4.0 are listed.

Putting the maximum transmit power limit (*P*_T_) defined by the regulations [48,49], the receiver sensitivity of the AT86RF212B transceiver [46], and the typical Bluetooth receiver sensitivity [51], peak realized gain of the transmitting and receiving antennas in Equation (2), the maximum communication range in free-space was calculated. This numerical calculation assumes the transmit antenna gain at 2.45 GHz to be 0 dBi, and the receiver antenna gains at both 868 MHz and 2.45 GHz to be 0 dBi. Using the parameter values listed in Table 1, the theoretical maximum communication range in free-space was calculated as 2333 m at 868 MHz and 456 m at 2.45 GHz. This calculation suggests that in comparison to 2.45 GHz, a higher communication range is possible to achieve at 868 MHz.

## 3. System Design

This section presents a detailed description of the developed wristwatch sensor platform. First, the hardware and the wireless software architecture of the sensor platform are discussed. Further, the working principle of a PPG sensor has also been reported.

### 3.1. System Hardware Architecture

**Figure 2:**
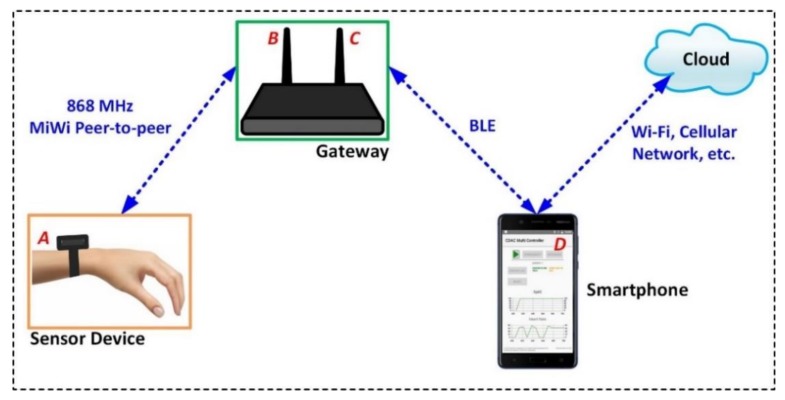
System hardware architecture.

The hardware architecture of the wrist-worn wireless sensor platform is depicted in Figure 2. The system hardware architecture broadly consists of four major constituents, denoted as a wristwatch, gateway, smartphone app, and cloud. The sensor device incorporates an SpO_2_ and an HR sensor and communicates with the gateway using an 868 MHz MiWi network protocol [52]. The gateway sends the sensed data to a smartphone over Bluetooth. The use of a smartphone is not a necessary requirement. However, as the smartphone app was already available with the research team, it was decided to use the smartphone application in the first stage instead of developing a new back end system to handle the data. This decision was taken for the following reasons: (a) the primary objective of the research focuses on the sensor to gateway communications, and (b) for the clinical trial, it was much easier for clinicians to record data directly from the mobile phone device.

**Figure 3:**
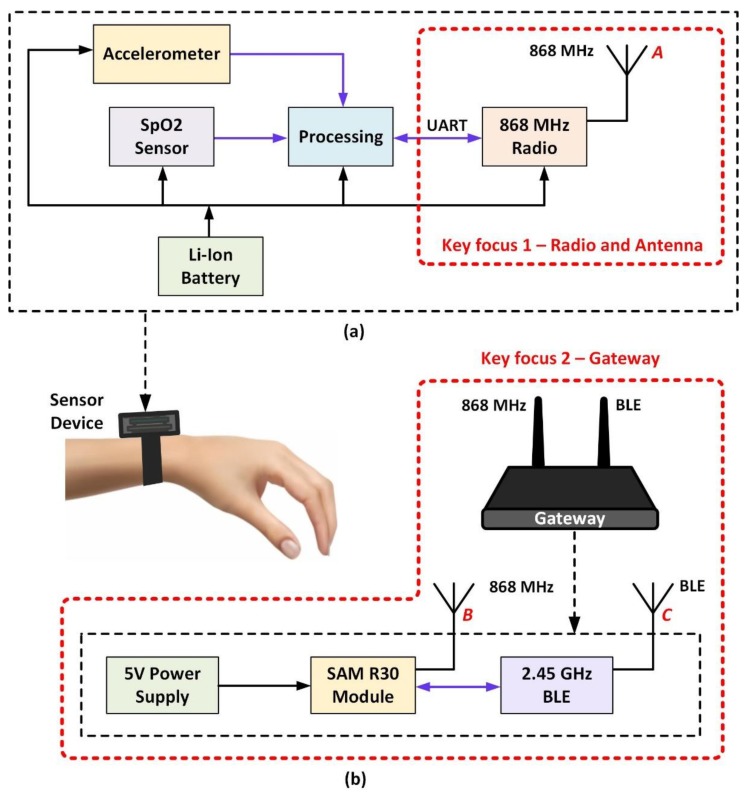
Internal sub-systems of the (**a**) wristwatch sensor device, (**b**) gateway.

Further, the data from the smartphone can be forwarded to the IoT cloud using any phone-supported cellular network such as Wi-Fi. In Figure 3a, the internal components of the wristwatch device, including a Li-ion battery, accelerometer, SpO_2_ sensor, processor, and an 868 MHz radio unit are illustrated. The radio unit uses an ATSAMR30E18A microcontroller (MCU), which has an integrated ultra-low power Sub-GHz band AT86RF212B transceiver [46]. 

When moved by a user, the wristwatch-integrated accelerometer wakes up the radio and processing units. Further, the data generated by the SpO_2_ and heart rate sensor are sent to the processor. The processed data are then forwarded to an 868 MHz radio transceiver. The radio transceiver feeds an 868 MHz wristwatch-integrated antenna, denoted as *A*, which transmits the processed data. In Figure 3b, the internal components of the gateway are illustrated. The gateway incorporates an 868 MHz whip antenna (denoted as *B*), which receives the data transmitted by the wristwatch sensor device. The implemented AT86RF212B transceiver provides a complete radio interface between the microcontroller and the antenna. This transceiver supports various modulation techniques such as binary phase-shift keying (BPSK) with a data rate of 20 and 40 kbps, and an offset quadrature phase-shift keying (O-QPSK) with a data rate ranging from 100 kbps to 1 Mbps. When compared to the O-QPSK modulation technique, BPSK is robust, simple to implement, and requires less power [53,54]. The modulation technique and the data rate are related to the receiver sensitivity, and the BPSK with a data rate of 20 kbps provides the best receiver sensitivity (−110 dBm) for the given radio. 

In this work, the BPSK modulation technique with a data rate of 20 kbps is implemented, which is appropriate for the targeted application that requires a throughput of 20 bytes every 3 s. The antenna *B* is connected to an ultra-low power SAM R30 hardware platform [55]. The SAM R30 module is powered by a 5V power supply, as shown. An nRF52 Bluetooth low energy (BLE) module is connected to the SAM R30 platform, which has an integrated 2.45 GHz antenna, denoted as *C* [56].

The antenna *C* forwards the sensor data, which is received by a smartphone antenna, denoted as *D* in Figure 2. In this way, the SpO_2_ sensor data can be accessed on a smartphone app. As depicted in Figure 3, the work in this paper mainly focuses on the development of an 868 MHz radio and antenna, and a gateway to establish the wireless communication between the wristwatch sensor platform and a smartphone app. Moreover, the sensor construction, data processing, and algorithms were provided by the industrial partner, and are considered beyond the scope of this work. 

### 3.2. System Software Design

The developed wristwatch sensor platform makes use of several software architectures at sensing, processing, and wireless connectivity levels. However, this work focuses only on the wireless software architecture aspect of the sensor platform. In this section, the implemented wireless network protocol and the system workflow is presented. 

#### 3.2.1. Wireless Network Protocol

In this work, the MiWi network protocol in the Sub-GHz band radio is implemented. MiWi is Microchip’s wireless protocol, which uses a low data rate and low power radio, based on IEEE 802.15.4 networks [52]. The proposed wristwatch sensor platform needs to satisfy the following requirements:

1. Bidirectional communication;
2. Minimum throughput that allows the reliable transfer of sensor data and control commands;
3. Handle a burst of data of 500 bytes;
4. Determine sleep/active cycles of the wristwatch to enable power saving.

To satisfy the abovementioned requirements and enable a continuous transmission of raw sensor data, a feasibility test was carried out with different wireless protocol configurations, such as with and without the MiWi protocol stack. For this purpose, two SAM R30 evaluation platforms from Microchip were used, and the observations of the four test cases are summarized in Table 2. 

First, the throughput test was carried out without using the MiWi protocol; however, a Microchip performance analyzer tool [57] for the SAM R30 platform was employed. In this experiment, a throughput of 25.84 kbps was achieved; however, this configuration does not have an option to explore data encryption security. Secondly, using the MiWi (version-6.2) protocol with transmit (Tx) only, reliable communication with a transmission throughput of 19.13 kbps was attained. In this test, the encryption security option was disabled. This experiment was performed to compute the throughput for similar conditions, with and without the MiWi protocol. 

In healthcare applications, data encryption security is a critical concern. Thus, in the next two experiments, the MiWi security option was enabled. In the first scenario, when the security option was enabled, and only Tx was used, a maximum one-way transmission throughput of 9.60 kbps was achieved. Further, when the radio receiver (Rx) and the transmitter (Tx) were used at the same time, and the security was enabled, the maximum throughput of 1.44 kbps was achieved. This throughput is sufficient to transmit the HR, SpO_2_ and control command data. The results of this test verify that the bidirectional communication between the wristwatch and the gateway with the minimum throughput requirements was achieved.

It was considered that the radio board is receiving the data from the processing board at a data rate of 115.20 kbps. However, with MiWi security enabled, the radio board can transmit the data at a maximum throughput of 9.60 kbps. Thus, a method was needed to manage the required burst of data. This requirement was fulfilled by saving the data that were being received from the processing board to an array, and transmitting the data through the wireless link only when the protocol stack was ready for transmission. This solution enables successful handling of bursts of data of 500 bytes. Further, an acknowledgement from the gateway was used to toggle an I/O pin of the radio board, which is used to inform the processing board when the radio board is not connected. Therefore, the processing board can put the watch into sleep-mode for the purpose of power saving. 

#### 3.2.2. System Workflow

After satisfying the requirements for the wristwatch sensor platform, the application code and the MiWi protocol were deployed in two devices, as discussed in the previous Section 3.2.1. 

**Figure 4:**
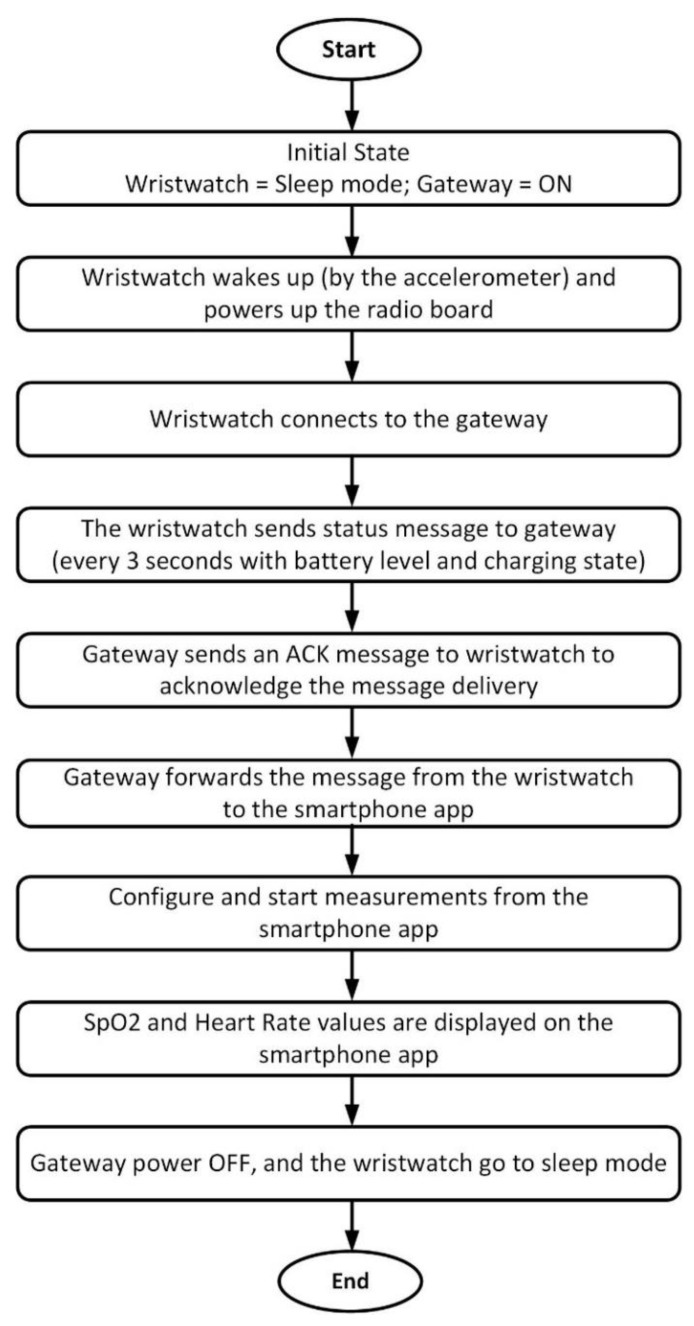
System workflow diagram.

One version is executed in the radio module of the wristwatch, and the second version was executed in the radio module of the gateway. In both the devices, the application-specific code, and the network protocol code reside within the same microcontroller. In Figure 4, the workflow diagram of the system is illustrated. In the initial state, the wristwatch sensor device is in the sleep-mode. The gateway is assumed to be powered ON in the initial state of the process. The wristwatch wakes up when a movement is detected by the accelerometer embedded in the processing board. At this stage, the processing board powers up the radio board, and the wristwatch connects to the gateway. Now, the wristwatch sends a status message with the battery level and charging state of the wristwatch to the gateway every three seconds. When the status message is received at the gateway, the gateway sends an acknowledgement (ACK) to the wristwatch. Further, the gateway forwards the message from the wristwatch to the smartphone application (user interface). Concurrently, the end-user can send the control commands from the smartphone to configure (measurement duration and patient ID), to start, or to stop a measurement.

To reduce human error, the patient ID is assigned using a patient-specific QR code. At this point, the SpO_2_ and HR measurements are completed, and the measured values are displayed on the smartphone interface. At the same time, the raw data were stored in a wristwatch-integrated SD card. Next, the gateway is turned OFF, and the wristwatch will go back to sleep-mode. In this way, the SpO_2_ and HR measurements of a patient in a clinical setting are accomplished, and the test setup becomes available for the next measurements. 

### 3.3. Working Principle of the PPG Sensor 

In this section, the working principle of a wrist-worn photoplethysmography (PPG) sensor is outlined. PPG is a key technique that uses optical sensors for non-invasive monitoring of the volume changes in the arterial blood [7]. Conventionally, an optical biosensor is used to measure the arterial oxygen saturation (SpO_2_) level. This method involves an inconvenient step, where a probe needs to be mounted on the finger while taking the measurements [8]. This work focuses on the development of a wristwatch-integrated wearable sensor device, because a wristwatch is easy and comfortable to wear for a long time and during various physical activities. 

**Figure 5:**
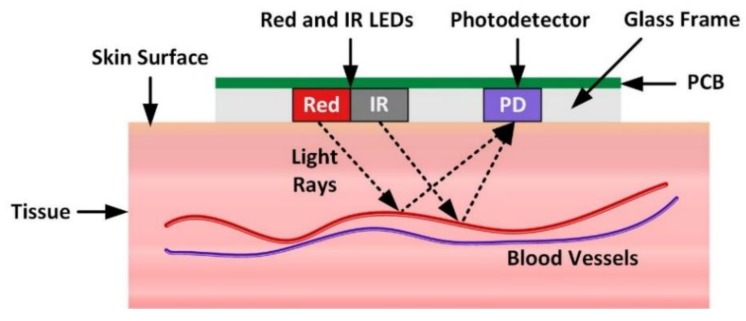
Working principle of a reflection-type PPG sensor.

PPG is widely used in pulse oximetry for SpO_2_ and heart rate measurements [9]. There are two modes of operation of a pulse oximeter: transmittance mode and reflectance mode. In transmission mode, the tissue sample is placed between the source light and the photodetector, while, in reflection mode, the LED and photodetectors are placed side-by-side. This work uses reflection mode pulse oximetry because of its flexibility and suitability for wearable sensors [7,8]. In Figure 5, the working principle of a reflection-type PPG sensor is illustrated. For SpO_2_ measurements, a PPG optical sensor utilizes two types of light sources (LED) to illuminate the tissue. The two commonly used light sources are red and infrared (IR), with respective wavelengths of 660 and 940 nm. The red and IR wavelengths give different absorption properties for oxygenated haemoglobin (HbO2) and haemoglobin (Hb). The captured light at the photodetector is subsequently analyzed to estimate the blood volume changes, based on the difference in the absorption of red and IR wavelengths. 

As shown in Figure 5, all the electronic components, such as LEDs and PD, are populated on a printed circuit board (PCB) and encased in a glass frame, as shown. The pulse signal obtained from a PPG sensor comprises an AC (pulsatile) and a DC (slowly varying) component. The AC component is attributed to changes in the blood volume synchronous with each heartbeat, whereas the DC component is related to respiration, tissues, and average blood volume [8].

The AC pulse signal is superimposed on the DC signal, where more than 90% of the pulse amplitude is contributed by the DC component [58]. Both the AC and DC waveforms are extracted using suitable filters and amplifiers, and later the AC waveforms are used for subsequent pulse analysis in a software tool like Matlab. Moreover, SpO_2_ is a demonstrator example of the PPG technology, and there are additional applications where PPG can be used for sensing other biological parameters (including blood pressure, respiration rate and anaesthetic depth) at different wavelengths [10]. 

## 4. Antenna Design

**Figure 6:**
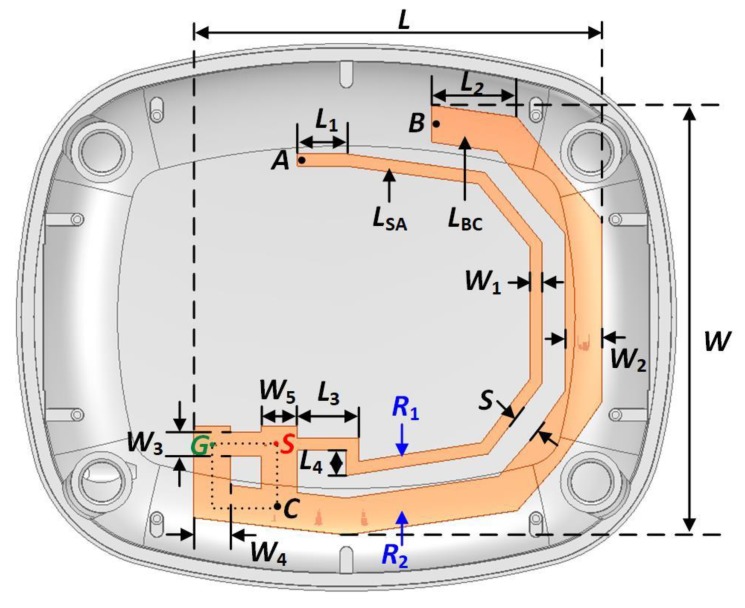
Antenna topology.

This section presents the design, fabrication and measurement of a compact 868 MHz wristwatch-integrated antenna. The design of a compact sub-GHz antenna to fit into a small area contained within a wristwatch device is challenging due to size constraints. In this case, the challenge is to integrate an antenna with a guided electrical length of 57 mm or approximately *λ*_g_/4 in a wristwatch device. The key performance parameters of the antenna, such as gain, efficiency, and bandwidth are fundamentally limited by the antenna size [59]. The geometry of the proposed antenna is shown in Figure 6. The antenna has a modified planar inverted-F (PIFA) topology. The antenna is excited at point *S* using an unbalanced feed with respect to the ground signal at point *G*. The signal is applied from the radio board using two contact springs at points *S* and *G*. The antenna topology is comprised of two resonators, *R*_1_ and *R*_2_, for improving the radiation performance of the antenna. The particular shapes of *R*_1_ and *R*_2_ were designed in order to keep the resonators away from the internal conducting components of the wristwatch device. An inductive shunt section between points *S* and *G* has been used to improve the impedance matching of the antenna by compensating the capacitance of the resonating arms *R*_1_ and *R*_2_ that are in close proximity to the ground plane. 

The overall antenna structure is printed on the surface of a wristwatch enclosure using the LDS method. The wristwatch enclosure is made of an acrylonitrile styrene acrylate (ASA) material, which has a measured relative permittivity of *ε*_r_ = 2.9 and a loss tangent of tan*δ* = 0.033 at 868 MHz. The electrical properties of all the dielectric materials were measured using a dielectric assessment kit (DAK) from Speag [60]. The maximum dimension of the antenna is 35.2 × 33.4 mm, and the total dimension of the wristwatch enclosure case is 53.9 × 44.8 × 17 mm. 

**Figure 7:**
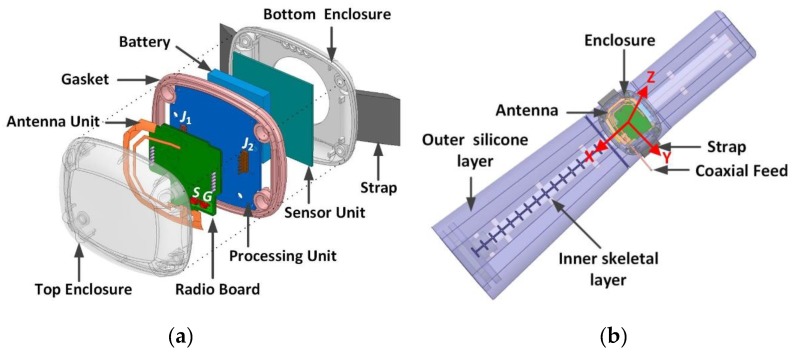
Wristwatch device model: (**a**) exploded view, (**b**) on a Speag phantom arm [60].

Figure 7a shows the exploded view of the wristwatch case model used in the electromagnetic (EM) simulations. The wristwatch case includes all the internal components of the device, such as a sensor unit, processing unit, radio board, and connectors. For the demonstrated system, the wristwatch casing is a modified, off-the-shelf assembly and is IP-65 rated [61], and contains an integrated rubber gasket between the bottom and bottom enclosure assemblies. The rubber gasket ensures a waterproof seal between the top and bottom enclosure. The connectors J_1_ and J_2_ join the processing unit to the sensor unit and the radio board. The contact springs denoted as *S* and *G* on the radio board, as shown in Figure 7a, connect respectively to the points *S* and *G* on the antenna structure, as shown in Figure 6. The simulation model, where the wristwatch device is placed on a commercial SHO-GFPC-V1 phantom arm from Speag [62], is shown in Figure 7b.

The phantom arm model is comprised of two layers, an outer silicone layer, and an inner skeletal layer. The outer silicone layer has *ε*_r_ = 30 and an electrical conductivity of *σ* = 0.7 S/m, and a mass density of 1200 kg/m^3^ at 868 MHz. The inner skeletal layer has *ε*_r_ = 30 and *σ* = 2.5 S/m at 868 MHz.

### 4.1. Antenna Simulations

In this section, the simulation results of the proposed antenna are summarized. The antenna was simulated using ANSYS full-wave high frequency structure simulator (HFSS) [63]. A parametric analysis of the length of the resonators *R*_1_ and *R*_2_ was performed in order to optimize the antenna for 868 MHz operations. It can be seen from Figure 6 that the parameter *L*_1_ determines the total length of the resonator *R*_1_, and *L*_2_ determines the total length of the resonator *R*_2_. It is shown in Figure 6 that the resonator *R*_1_ extends between points S and A, and has a total length (*L*_SA_) of 57 mm. This value of *L*_SA_ corresponds to a guided electrical length of 0.28 *λ*_g_, which is close to *λ*_g_/4 at 868 MHz. 

**Figure 8:**
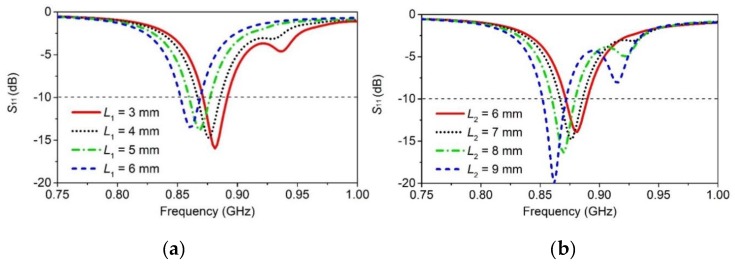
Simulated *S*_11_ response: (**a**) for varying L_1_, (**b**) for varying L_2_.

The resonator *R*_2_ has a total length of *L*_BC_ = 60 mm, which corresponds to an estimated guided electrical length of 0.29 λ_g_ at 868 MHz. The guided wavelength was calculated using the relative permittivity (*ε*_r_ = 2.9) of the wristwatch enclosure, which is in close proximity to the resonators. As shown in Figure 8, the value of *L*_1_ was varied between 3 to 6 mm, and the value of *L*_2_ was varied between 6 to 9 mm. The parameters *L*_1_ and *L*_2_ mainly control the resonant frequency of the antenna, and as expected, increasing the values of either *L*_1_ or *L*_2_, the resonant frequency decreases continuously.

The parameters *L*_1_ and *L*_2_ also have effects on the impedance-matching of the antenna. However, in comparison to *L*_1_, *L*_2_ has more influence on the impedance matching. This is expected because the resonance around the 868 MHz band is contributed by the resonator *R*_2_. Thus, by adjusting the values of the parameters *L*_1_ and *L*_2_, the antenna can be tuned at the desired resonant frequency, and the impedance matching can be controlled to some extent. The antenna is resonant at 868 MHz for *L*_1_ = 4 mm and *L*_2_ = 7 mm. The final parameters of the proposed antenna are summarized in Table 3. 

**Figure 9:**
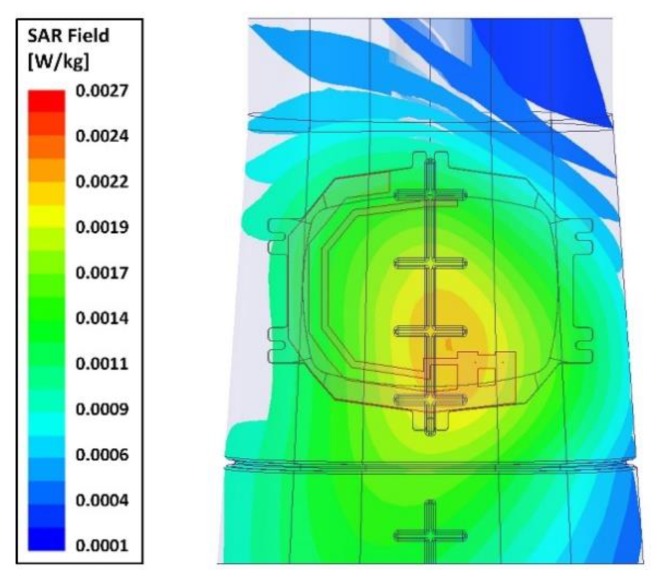
Simulated specific absorption rate (SAR) distribution on a Speag phantom arm [62] at 868 MHz.

In Figure 9, the simulated specific absorption rate (SAR) of the proposed wrist-worn antenna on a Speag phantom arm at 868 MHz is shown [62]. The simulation environment for the SAR evaluation of the antenna is illustrated in Figure 7b. The peak SAR point on the phantom arm is located near the feed point of the antenna. For an input power of 1 mW, a peak SAR of less than 0.003 W/kg was demonstrated. This figure is less than 0.1% of the maximum permissible SAR limit of 4 W/kg averaged over 10 grams of wrist tissue [64]. 

### 4.2. Impedance Matching and Bandwidth Enhancement

**Figure 10:**
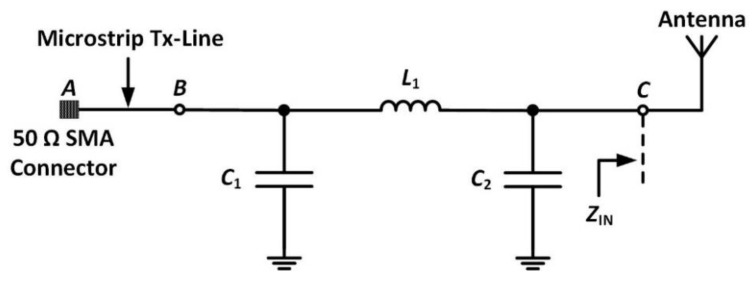
*Π*-type impedance-matching network.

The input impedance (*Z*_IN_) of an antenna is a key design parameter that determines the reflection coefficient and the impedance bandwidth of the antenna [65]. In this section, an impedance-matching network to reduce the antenna reflection coefficient while also improving the impedance bandwidth of the antenna is presented. The matching circuit shown in Figure 10 ensures the maximum power transfer from the source (*A*) to the antenna and maintains the desired performance even under small detuning effects [66]. First, a vector network analyzer [67] was used to measure *Z*_IN_, which was later used to design an optimal matching network for the antenna. 

As shown in Figure 10, a π-type matching network between points *B* and *C* has been implemented. The matching components include a shunt capacitor *C*_1_ on the source side, a series inductor *L*_1_, and a shunt capacitor *C*_2_ towards the load side. 

The π-type matching network matches the *Z*_IN_ of the antenna to a 50 Ω SMA connector at point *A*, as shown. The values of the matching network components were optimized using AWR Microwave Office [68]. The matching network optimization was performed to reduce the power reflection at the input port of the antenna by varying the values of the circuit components *C*_1_, *C*_2_ and *L*_1_. The final realized values of the matching components are listed in Table 4.

### 4.3. Antenna Prototype Fabrication 

**Figure 11:**
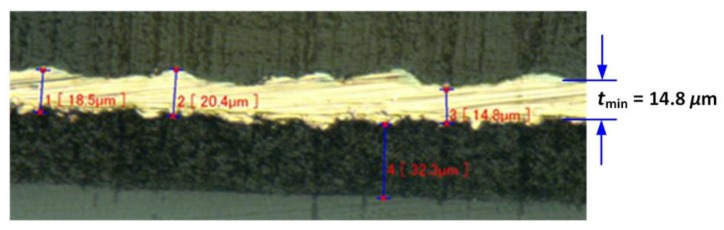
Metallization of the antenna track.

The proposed antenna was fabricated using an LPKF laser direct structuring (LDS) Protolaser 3D System, available within the Tyndall Microsystems Packaging Laboratory [71]. For antenna fabrication, the LDS technology was chosen because of its advantages such as low cost, easy integration to the enclosure structure, and no need for additional flex PCB [72,73]. The fabrication of the proposed antenna prototype using the LDS technology was completed in the following steps:

1. 3D part fabrication or selection: The 3D part/object on which the metal has to be printed can be fabricated using standard 3D printers. The commercially available thermoplastic parts, such as metals, plastics, glass, FR4, can also be used. In this work, the antenna structure is printed on an ASA thermoplastic wristwatch enclosure from OKW enclosures [61];
2. Part coating with ProtoPaint epoxy: The part is covered with the LPKF ProtoPaint LDS epoxy layer;
3. Laser direct structuring: The LPKF laser system creates an outline of the conductive pattern of the design. In this step, the laser removes some of the epoxy material and forms a rough surface on which the copper can firmly adhere during metallization;
4. Metallization: This step involves the electroless copper plating of the region exposed by laser etching. The photograph of the antenna track during the metallization process is shown in Figure 11. The metallization of the antenna track was completed in the following four steps:
  - Step 1: In order to get a low resistance electrical continuity through the activated track on the plastic, copper electroless deposition of the surface was required to make it possible to electroplate it. Using an in-house developed, dimethylamine borane (DMAB)-based copper electroless deposition solution, the track was metallized with copper. The sample was immersed in the bath for 60 min at 70 °C, pH9;
  - Step 2: The electroless copper deposited on the sample needed to be electroplated up with copper. A Schlotter commercial copper bright bath, ACG8, was utilized for this process. The sample was plated for 60 min, 2 A/dm^2^ at room temperature;
  - Step 3: Utilizing an in-house developed, low stress nickel-sulphamate-based electroplating bath, the sample was plated for 10 min, 3 A/dm^2^, at 60 °C. The minimum thickness (*t*_min_) of the electroplated copper is 14.8 μm, as shown;
  - Step 4: To avoid oxidation of the nickel surface, a commercially available gold Ormex immersion solution by Engelhard was used to finish the surface with gold. The thickness of the gold finish is less than 0.11 μm. This process took 7 minutes at a temperature of 85 °C.

**Figure 12:**
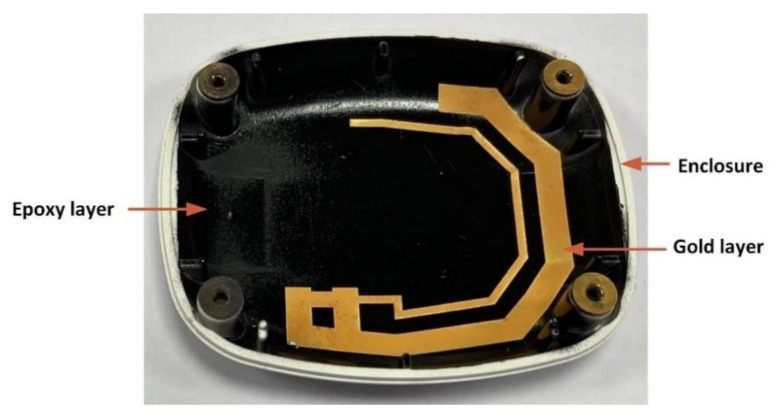
LPKF laser direct structuring (LDS) printed antenna.

The proposed antenna was fabricated using the abovementioned fabrication steps, and the final developed antenna prototype is shown in Figure 12.

### 4.4. Antenna Measurements

**Figure 13:**
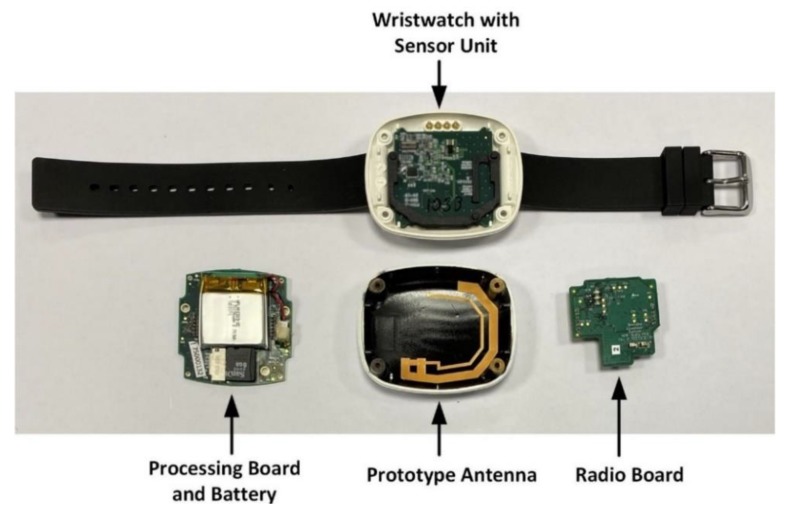
Wristwatch model assembly.

In this section, the measured impedance and radiation characteristics of the wristwatch-integrated antenna are described. In Figure 13, a photograph of the wristwatch model with an integrated sensor unit, radio board, processing board with an attached battery, and the prototype antenna is illustrated. The Li-ion rechargeable battery used in this work has a voltage level of 3.7 V and a current level of 190 mAh [74]. For clear visibility, a 3D stack-up of the wristwatch device has already been illustrated in Figure 7a.

**Figure 14:**
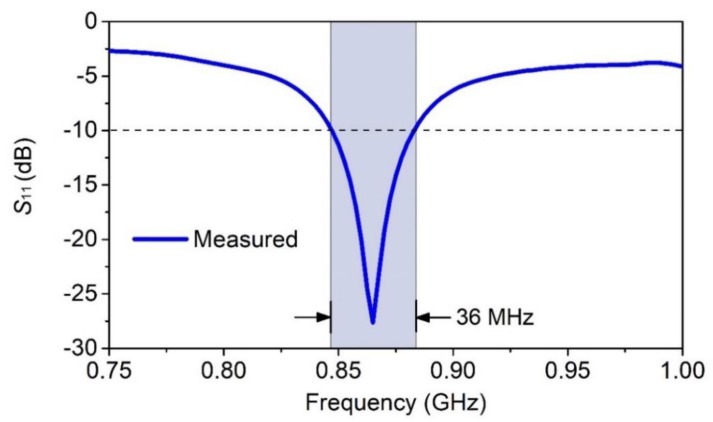
Measured *S*_11_ response of the antenna.

The measurements of the wristwatch-integrated antenna were performed after placing the wristwatch device on a commercial SHO-GFPC-V1 phantom arm [62]. First, the impedance characteristic of the antenna was measured using a Rohde and Schwarz ZVRE vector network analyzer (VNA) [67]. In Figure 14, the measured *S*_11_ response of the antenna is illustrated. As discussed earlier, in order to improve the impedance matching and the impedance bandwidth of the antenna, a π-type matching network was implemented. The matching network improves the |*S*_11_| and enables a wideband-matched response around the 868 MHz band. The measured prototype antenna has a −10 dB impedance bandwidth of 36 MHz. This figure is five times higher than the minimum required bandwidth specification of 7 MHz (863–870 MHz) at the 868 MHz band [75].

**Figure 15:**
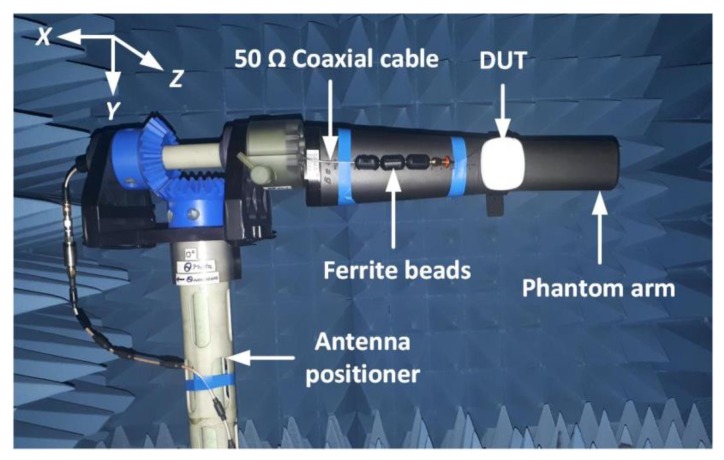
Device under test (DUT) measurement setup in AMS-8050 anechoic chamber [76].

Secondly, the 3D radiation characteristics of the wristwatch device under test (DUT) was measured in an AMS-8050 antenna measurement system [76]. Figure 15 illustrates the anechoic chamber measurement setup of the DUT. The rotation of the DUT in the chamber is controlled using the multi-axis positioning system (MAPS) or simply the antenna positioner, as shown. The wristwatch device has been placed on an SHO-GFPC-V1 phantom arm [62] and is supported on a mounting fixture, as shown. 

The antenna is excited using an unbalanced 50 Ω coaxial cable. As the connecting cable can affect the antenna properties, multiple ferrite beads have been incorporated in the measurement setup to minimize the effect of the cables on the antenna properties. To illustrate the reliability of the measurement setup, a 2D radiation pattern of a reference 915 MHz antenna that was characterized in a similar chamber setup is shown in Supplementary Figure S1, where a close agreement between simulated and measured results is demonstrated.

**Figure 16:**
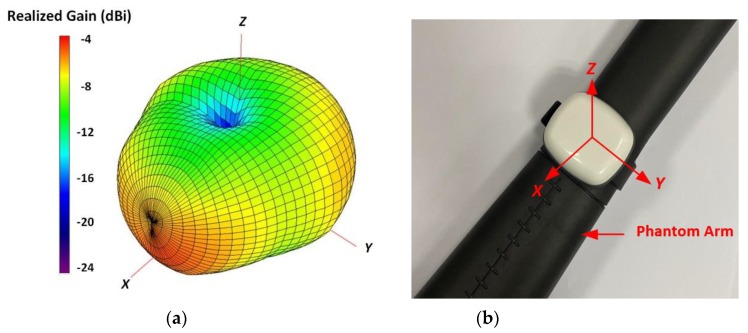
(**a**) Measured antenna realized gain (3D) at 868 MHz, (**b**) coordinate system of the wristwatch device placed on a phantom arm.

In Figure 16a, the measured 3D realized gain pattern of the developed antenna at 868 MHz is shown. The coordinate system of the wristwatch device, placed on a phantom arm, is also shown in Figure 16b. In the *xy*-plane, the antenna is characterized as a typical dipole-like radiation characteristic with an omnidirectional radiation pattern. The antenna exhibits nulls along the *z*-axis in the *yz* and *xz*-planes. These radiation nulls are expected due to the shielding effect of the finite PIFA ground plane and the absorption of the radiated energy by the phantom arm, along the *z*-axis.

The on-body prototype antenna exhibits a measured peak realized gain of −4.86 dBi and a radiation efficiency of 14.53 % at 868 MHz. To summarize the antenna design aspects, contrary to the Sub-GHz band wrist-worn antennas reported in the literature [35,36,37,38,39], the antenna designed in this work includes all the internal components within a practical wristwatch wireless device and the measured results exhibit practically acceptable impedance and radiation characteristics at 868 MHz.

## 5. Communication Range Measurements of the Sensor Platform

**Figure 17:**
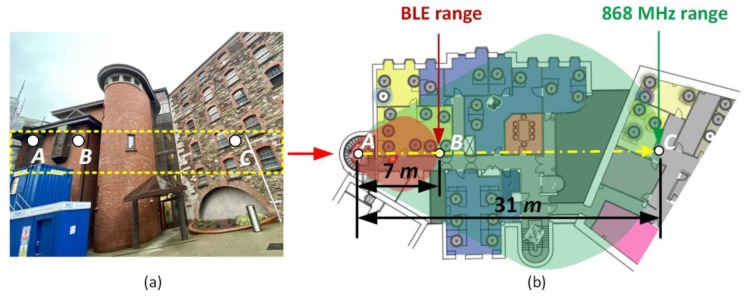
Indoor wireless communication range measurement (**a**) front view of the building, (**b**) 2D map of the floor where measurements were performed.

To evaluate the performance of the developed 868 MHz wireless sensor platform, communication range measurements were conducted in an indoor environment, as shown in Figure 17. In addition, for the purpose of comparison, the communication range measurement for a commercial BLE wristwatch device is also performed [77]. The indoor environment represents a typical office structure and includes several clutters between the transmitter and the receiver, such as thick and old concrete walls, wooden cubicles, PCSs, tables, chairs and closets. 

Figure 17a shows the front view of the office building where the indoor range measurements were performed. The measurements were taken on the second floor of the building, and for clarity, the 2D map of the floor is illustrated in Figure 17b. The measurements were conducted after fixing the gateway at point *A*, and the position of the wristwatch sensor platform was changed in steps inside the building. In Figure 17, the point *C* represents the edge of the communication at 868 MHz. It is important to mention that the walls between the transmitter (wristwatch sensor platform) and the receiver (gateway) act as an attenuator and negatively affect the wireless communication range. 

Typically, in the normal mode, the AT86RF212B transceiver transmits at a power level of 5 dBm [46]. For the communication range measurements of the 868 MHz sensor platform, the power level of both the transmitter and the receiver was set at 5 dBm and the receiver sensitivity at −110 dBm. For the given indoor scenario, a maximum communication range between points *A* and C of approximately 31 m was demonstrated.

In this work, a sensor platform operating at only 868 MHz was developed, and for the purpose of comparison, the BLE range of a Fitbit Charge 3 smartwatch in the same indoor environment was evaluated [77]. Point *B* in Figure 17 represents the communication edge of the BLE Fitbit Charge 3 smartwatch, which is approximately 7 m from point *A*.

Thus, from the range measurements of the developed sensor platform, and its comparison with the Fitbit Charge 3 BLE, it is observed that an improved communication range is possible at 868 MHz. This study shows that when compared to the smartwatch BLE device, communications at 868 MHz offers more than quadruple communication range and is more suitable for indoor applications, such as in offices and hospitals.

## 6. System Implementation and Clinical Trials 

In this section, the implementation of the developed wristwatch wireless sensor platform and the clinical trial results are summarized. The clinical trials were performed at Mercy University Hospital, Cork, Ireland [78]. “The patients gave their informed consent for inclusion before they participated in the study. The study was conducted in accordance with the Declaration of University College Cork, Cork, Ireland, and has been approved by the Clinical Research Ethics Committee (ECM 4 (a) 07/05/19 and ECM 3 (IIIII) 28/06/19)”. The trials using the developed sensor platform were successfully conducted on 24 patients over a period of four weeks, and under the supervision of clinical staff. Each trial takes approximately 3 minutes to measure the SpO_2_ and heart rate of a patient. 

To access the SpO_2_ and heart rate measurement data on a smartphone application, a gateway was developed, as illustrated in Supplementary Figure S2. The gateway enclosure has a dimension of 150 × 100 × 45 mm and is made of acrylonitrile butadiene styrene (ABS) material [79]. The internal components of the gateway include a Microchip SAM R30 Xplained Pro hardware platform [55], an 868 MHz whip antenna, a 2.45 GHz (Bluetooth low energy) BLE module [56], two types of LEDs [80,81], a 5 V USB power port, and some cables for internal connections. The 868 MHz wristwatch-integrated radio communicates wirelessly with the whip antenna. Initially, both the LEDs (green and blue) remain OFF. When the gateway is powered up by a 5 V supply, the green LED turns ON, indicating that the gateway is ready for the communication. When the blue LED starts blinking, it shows that a successful connection has been established. The nRF52 BLE module is connected to the SAM R30 platform, which has an integrated 2.45 GHz chip antenna [56]. The BLE antenna transmits the sensor data, which can be received by a smartphone app. The sensor data on the smartphone app could be placed on the IoT-cloud and, when required, an authorized user can easily access the sensor data using any phone-supported cellular network. 

**Figure 18:**
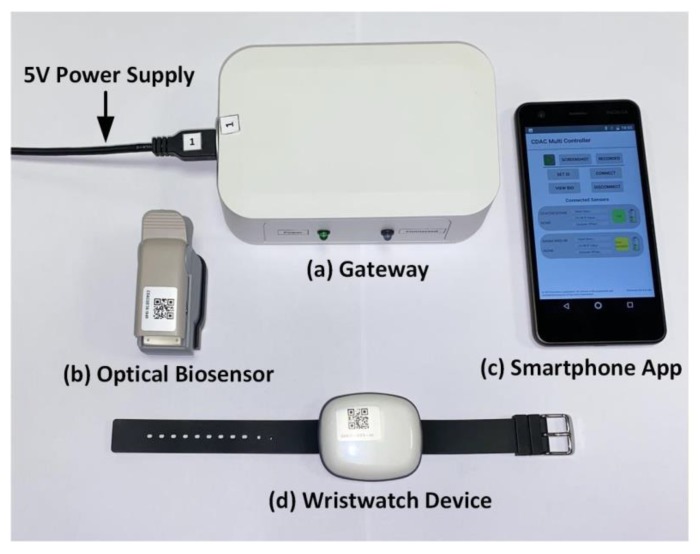
Clinical trial test devices.

**Figure 19:**
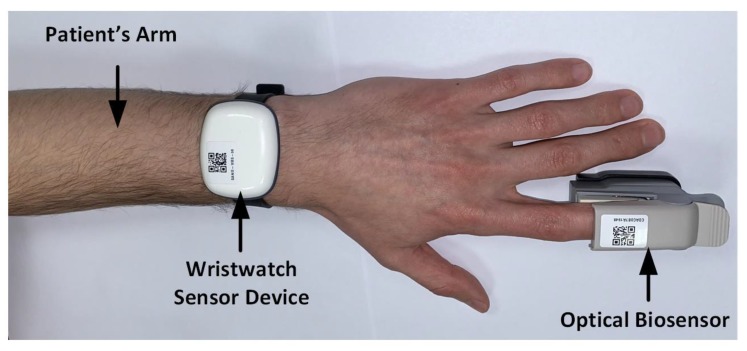
Wristwatch sensor device worn by a patient in a clinical setting.

In Figure 18, the devices used in the clinical trials are shown, such as a gateway, an optical biosensor, a smartphone, and the developed wristwatch sensor platform are illustrated. The gateway works as a communication link between the wristwatch sensor platform and the smartphone app. As shown, the clinical trials also employ an existing optical biosensor device, which can wirelessly connect to the smartphone app. Figure 19 illustrates the SpO_2_ and heart rate measurement setup on a patient’s arm using the developed wristwatch sensor device as well as the existing optical biosensor in a clinical setting. The test protocol used in the clinical trials specified that the arm be parallel to the ground, as the patient would usually be sitting or lying down. This position is recommended because the PPG devices are heavily impacted by motion artefacts, which are likely to have a significant influence on the quality of the measured data [82,83,84]. 

**Figure 20:**
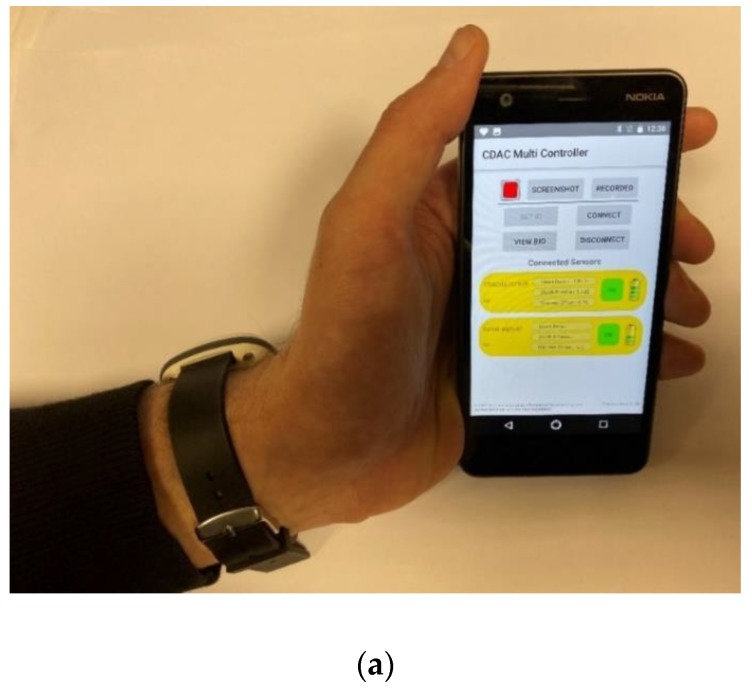

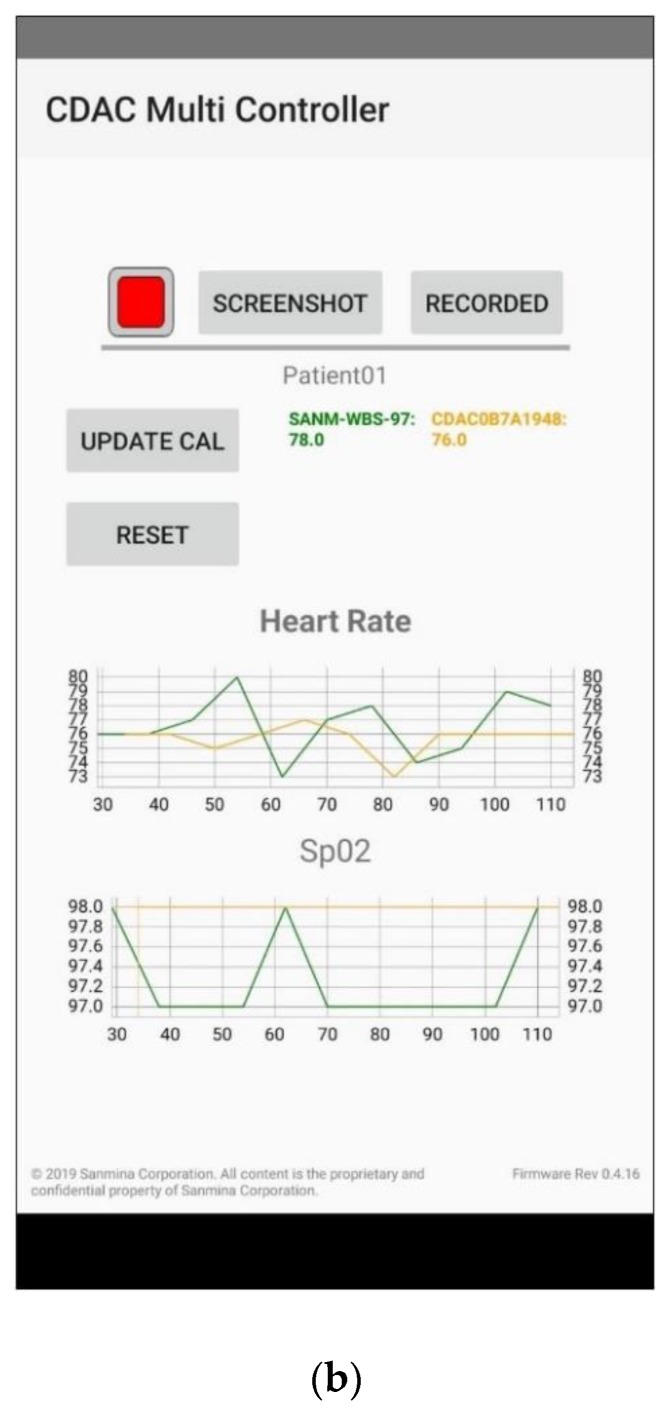
Smartphone application for SpO_2_ and heart rate measurements (**a**) smartphone app in a user’s hand, (**b**) screenshot of the measured plots.

The sensor data measured by the wristwatch device were compared against the optical biosensor data. Figure 20a shows the smartphone app in a user’s hand, showing that both the sensor devices (wristwatch sensor and optical biosensor) are connected. The battery status of the wristwatch and the optical biosensor device can be seen on the app. When both the sensors are placed on the patient’s arm (as shown in Figure 19) and the patient ID is set using QR codes, the setup is ready for the measurements. The plots of the measured SpO_2_ and HR sensor data on the smartphone app are shown in Figure 20b. The green curves represent the SpO_2_- and HR-measured values by the wristwatch device, and the yellow curves represent the optical biosensor-measured data. It can be seen that the measurement results from the developed sensor wristwatch device are in close agreement with the results from the optical biosensor device.

## 7. Conclusions

This paper has presented the design and development of a novel wristwatch-based wireless sensor platform operating at the Sub-GHz (868 MHz) ISM band, with an example application demonstrated for arterial oxygen saturation (SpO_2_) and heart rate measurements. Based on numerical calculations and the results reported in the literature, it was shown that the integration of the 868 MHz transceiver offers several advantages such as less path loss, reduced wireless co-existence issues, less attenuation through materials, and improved wireless communication range. However, in contrast to 868 MHz, the 2.45 GHz ISM band is available worldwide, and at the higher frequency, a relatively compact antenna solution is possible. The working principle of a wrist-worn non-invasive optical photoplethysmography (PPG) sensor is also outlined. 

The hardware and the wireless software architecture of the sensor platform are proposed. The sensor device incorporates a SpO_2_ and heart rate sensor and communicates with a gateway using an 868 MHz MiWi network protocol. The MiWi wireless network protocol was implemented because of its potential to offer a low data rate, low power, and low complexity. The implemented radio transceiver uses BPSK modulation, operating with a data rate of 20 kbps, which is more than sufficient for the target application. 

The main contribution of this work is the development of a highly integrated 868 MHz wristwatch radio and antenna. The proposed antenna topology is a variant of a planar inverted-F antenna structure and is printed on the 3D surface of a wristwatch enclosure using laser direct structuring (LDS) technology. To improve the impedance matching and to enhance the impedance bandwidth, a π-type matching network was implemented. 

The measured on-body antenna exhibits a −10 dB impedance bandwidth of 36 MHz. In addition, the on-body antenna results show a peak realized gain of −4.86 dBi and a radiation efficiency of 14.53% at 868 MHz. Moreover, for 1 mW of input power, a simulated specific absorption rate (SAR) value of 0.003 W/kg at 868 MHz is demonstrated. This figure is less than 0.1% of the maximum permissible limit of 4 W/kg for wrist-worn devices. 

To evaluate the performance of the developed 868 MHz wireless sensor platform, the wireless communication range measurements were conducted in an indoor office environment. The measured results demonstrated a communication range of approximately 31 m for the 868 MHz sensor platform, which is approximately four times greater than the commercial Fitbit Charge 3 BLE wristwatch device. 

Furthermore, the implementation of the developed wristwatch sensor platform and the clinical trial results are performed. The design of the gateway, which is used to forward the wristwatch sensor data to a smartphone app, is also reported. The developed wrist-worn sensor platform was effectively implemented for SpO_2_ and heart rate measurements of the patients in a clinical setting. The successful clinical trials confirm the potential of the developed sensor platform in future wearable health monitoring IoT applications. The future research work will focus on eliminating the use of a smartphone so that the gateway can directly communicate with the cloud. 

## Figures and Tables

**Table 1:** Parameters for calculating the communication range at 868 MHz and BLE 4.0.

Parameters	868 MHz	BLE 4.0
Transmit power limit (dBm)	14 [48]	10 [49]
Receiver sensitivity (dBm)	−110 [46]	−95 [51]
Wristwatch *T*x antenna gain (dBi)	−4.86 (Measured)	0
Receiver antenna gain, assumed (dBi)	0	0
Maximum communication range, *d* (m)	2333	456

**Table 2:** Radio throughput under different network protocol stacks configurations.

Configurations	Throughput (kbps)	Comments
Without MiWi stack	25.84	No security option
Using MiWi, Tx only	19.13	Security Disabled
Using MiWi, Tx only	9.60	Security Enabled
Using MiWi (Both Tx and Rx at the same time)	1.44	Security Enabled

**Table 3:** Final parameters of the proposed antenna.

**Parameter**	*L*	*W*	*L*_SA_	*L*_BC_	*L*_1_	*L*_2_	*L*_3_
**Value (mm)**	35.2	33.4	57	59	4	7	5
**Parameter**	*L*_4_	*W*_1_	*W*_2_	*W*_3_	*W*_4_	*W*_5_	*S*
**Value (mm)**	3	1	3	2	3	3	2

**Table 4:** Summary of the used matching network components.

Matching Component	Value	Part Number
*C*_1_	6.8 pF	600L6R8BT200T [69]
*C*_2_	4.7 pF	600L4R7BT200T [69]
*L*_1_	8.2 nH	0402WL8R2 [70]

## Supplementary Materials

The following are available online at https://www.mdpi.com/1424-8220/20/6/1675/s1, Figure S1: Simulated and measured 2D radiation characteristics of the reference antenna at 915 MHz (a) xy-plane, (b) yz-plane, and (c) xz-plane, Figure S2: Internal components of the gateway.
