# Supplementary material for: A Wristwatch-Based Wireless Sensor Platform for IoT Health Monitoring Applications
Source: Sensors (Basel). 2020 Mar 17;20(6):1675. doi: 10.3390/s20061675 (PMC7147171; doi:10.3390/s20061675)
Supplement: Supplementary file 1 [file sensors-20-01675-s001.pdf]

sensors

# Supplementary Materials:

## A Wristwatch-Based Wireless Sensor Platform for IoT Health Monitoring Applications

**Sanjeev Kumar[1], John L. Buckley[1], John Barton[1], Melusine Pigeon[1], Robert Newberry[2], Matthew Rodencal[2], Adhurim Hajzeraj[1], Tim Hannon[2], Ken Rogers[1], Declan Casey[1], Donal O'Sullivan[1], Brendan O'Flynn[1]**

[1] Tyndall National Institute, University College Cork, Dyke Parade, T12R5CP, Cork, Ireland
[2] Sanmina Corporation, 13000 S. Memorial Parkway, Huntsville, AL 35803, USA
* Correspondence: sanjeev.kumar@tyndall.ie; Tel.: +353-212-346-109

**Supplementary Figure 1**

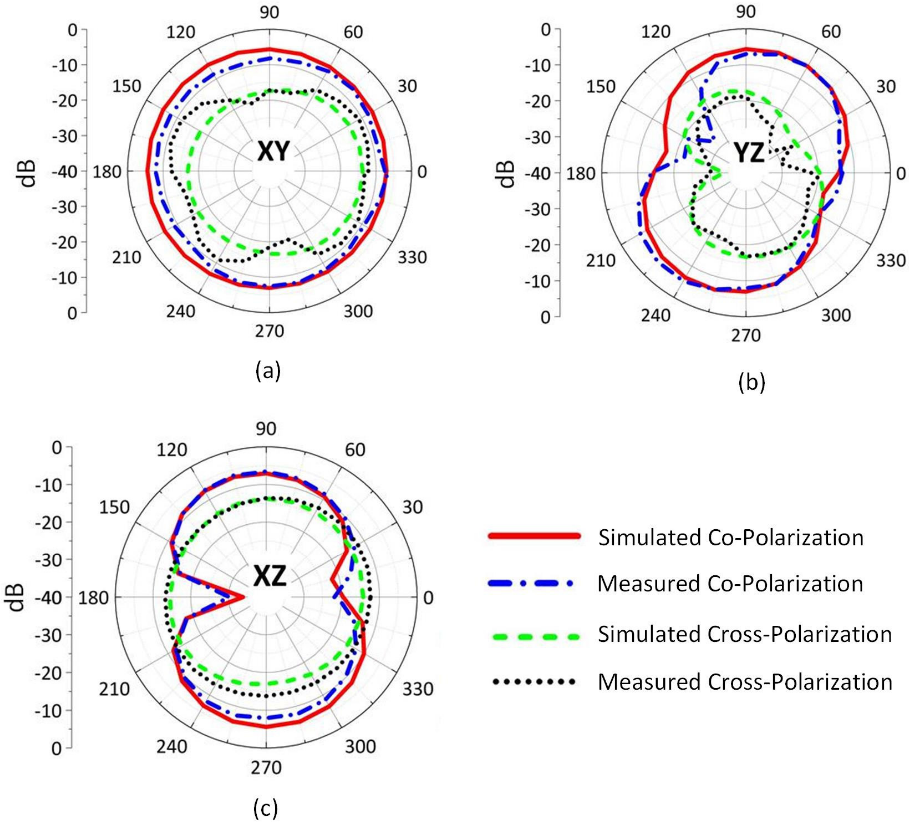


**Figure 1.** Simulated and measured 2D radiation characteristics of a reference antenna at 915 MHz (a) *xy*-plane, (b) *yz*-plane, and (c) *xz*-plane.

**Supplementary Figure 2**

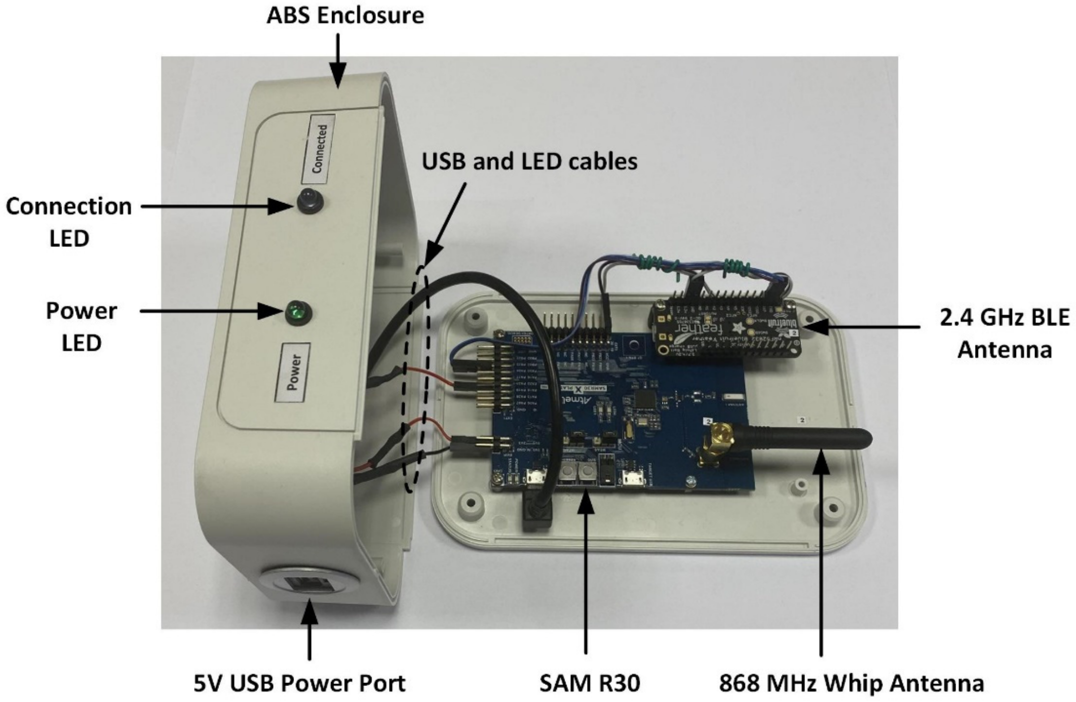


**Figure 2.** Internal components of the gateway.

© 2020 by the authors. Licensee MDPI, Basel, Switzerland. This article is an open access article distributed under the terms and conditions of the Creative Commons Attribution (CC BY) license (http://creativecommons.org/licenses/by/4.0/).
